# Correction: SRF-deficient astrocytes provide neuroprotection in mouse models of excitotoxicity and neurodegeneration

**DOI:** 10.7554/eLife.101107

**Published:** 2024-07-01

**Authors:** Surya Chandra Rao Thumu, Monika Jain, Sumitha Soman, Soumen Das, Vijaya Verma, Arnab Nandi, David H Gutmann, Balaji Jayaprakash, Deepak Nair, James P Clement, Swananda Marathe, Narendrakumar Ramanan

**Keywords:** Mouse

Thumu SCR, Jain M, Soman S, Das S, Verma V, Nandi A, Gutmann DH, Jayaprakash B, Nair D, Clement JP, Marathe S, Ramanan N. 2024. SRF-deficient astrocytes provide neuroprotection in mouse models of excitotoxicity and neurodegeneration. *eLife* **13**:e95577. doi: 10.7554/eLife.95577.

Published 30 January 2024

In the original version of the paper, image showing GFAP immunostaining for 12 mpi control neocortex in 1H is the same as GFAP immunostaining for 2 mpi control neocortex in 1F. We believe that this unfortunate error probably occurred while arranging the individual scaled images for the figure. We have now provided the correct image for 1H. We sincerely apologize for this oversight. There are no changes in the text.

The corrected Figure 1 is shown here:

**Figure 1:**
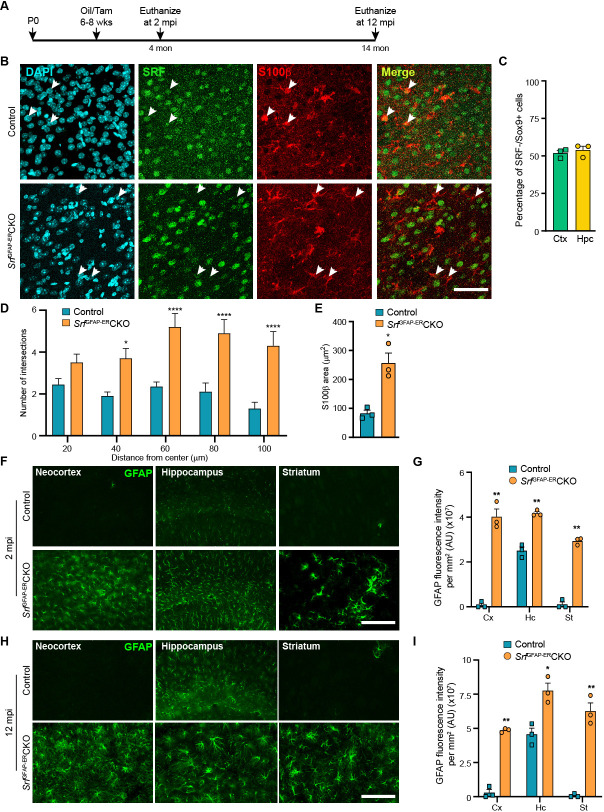


The originally published Figure 1 is shown for reference:

**Figure 2:**
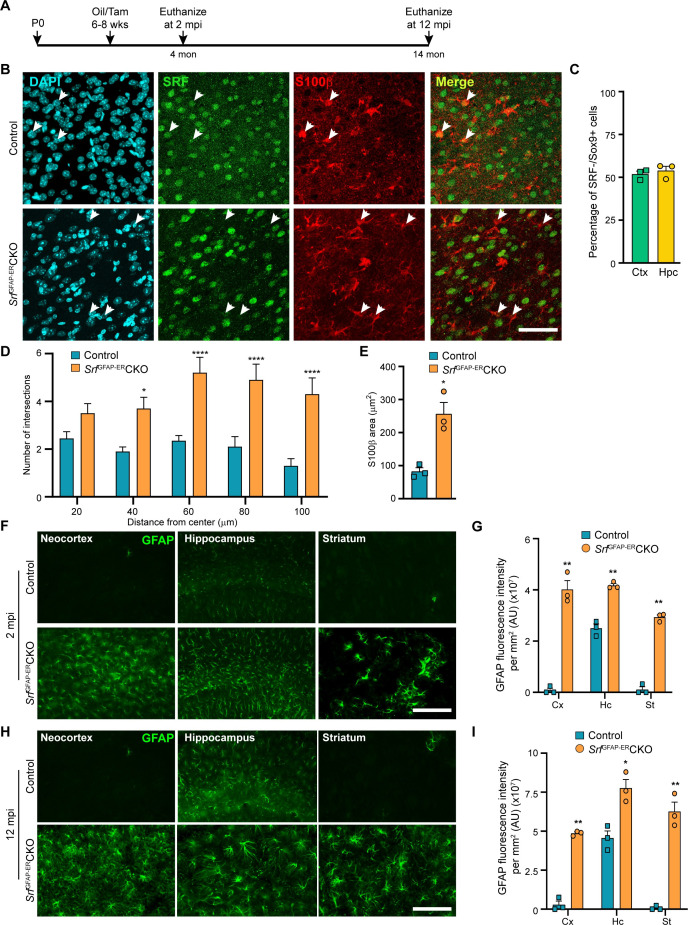


The article has been corrected accordingly.

