# Supplementary material for: SRF-deficient astrocytes provide neuroprotection in mouse models of excitotoxicity and neurodegeneration
Source: eLife. 2024 Feb 9;13:e95577. doi: 10.7554/eLife.95577 (PMC10857791; doi:10.7554/eLife.95577)
Supplement: Figure 5—source data 2. [file elife-95577-fig5-data2.docx]

**Table 2. Genes regulated in *Srf*^GFAP-ER^CKO astrocytes.**
