# Supplementary figures and images for: SRF-deficient astrocytes provide neuroprotection in mouse models of excitotoxicity and neurodegeneration
Source: eLife. 2024 Feb 9;13:e95577. doi: 10.7554/eLife.95577 (PMC10857791; doi:10.7554/eLife.95577)

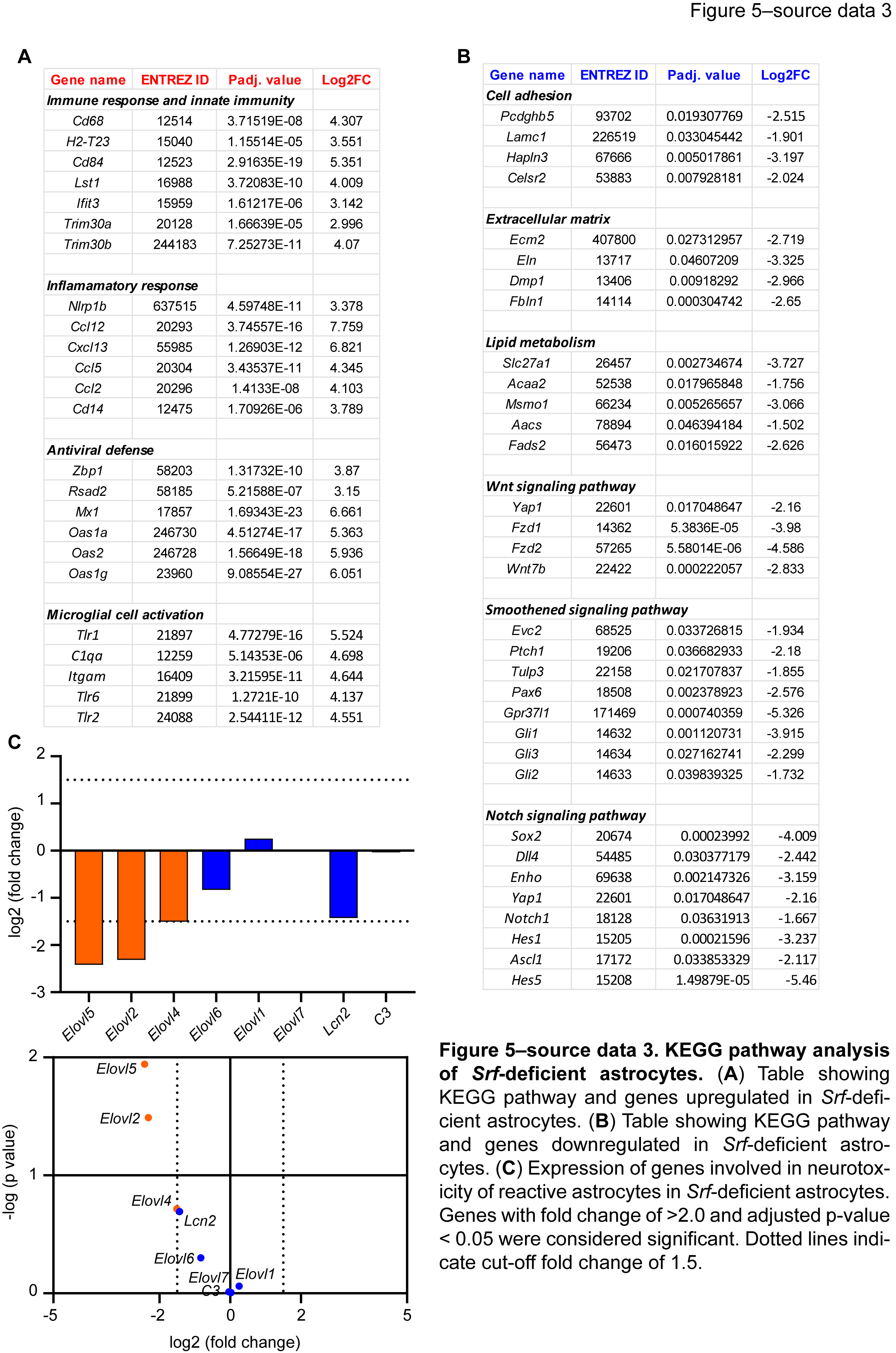

Supplement: Figure 5—source data 3. — (A) Table showing KEGG pathway and genes upregulated in Srf-deficient astrocytes. (B) Table showing KEGG pathway and genes downregulated in Srf-deficient astrocytes. (C) Expression of genes involved in neurotoxicity of reactive astrocytes in Srf-deficient astrocytes. Genes with fold change of >2.0 and adjusted p-value < 0.05 were considered significant. Dotted lines indicate cut-off fold change of 1.5. [file elife-95577-fig5-data3.zip › Figure_5-source_data3.tif]
